# The genome sequence of the Case-bearing Clothes moth, *Tinea pellionella *(Linnaeus, 1758)

**DOI:** 10.12688/wellcomeopenres.21015.1

**Published:** 2024-03-01

**Authors:** Douglas Boyes, Clare Boyes, University of Oxford and Wytham Woods Genome Acquisition Lab, Darwin Tree of Life Barcoding collective, Wellcome Sanger Institute Tree of Life Management, Samples and Laboratory team, Wellcome Sanger Institute Scientific Operations: Sequencing Operations, Wellcome Sanger Institute Tree of Life Core Informatics team, Tree of Life Core Informatics collective, Darwin Tree of Life Consortium

**Affiliations:** 1 UK Centre for Ecology & Hydrology, Wallingford, England, UK; 2 Independent researcher, Welshpool, Wales, UK

**Keywords:** Tinea pellionella, Case-bearing Clothes moth, genome sequence, chromosomal, Lepidoptera

## Abstract

We present a genome assembly from an individual female *Tinea pellionella* (the Case-bearing Clothes moth; Arthropoda; Insecta; Lepidoptera; Tineidae). The genome sequence is 245.3 megabases in span. Most of the assembly is scaffolded into 30 chromosomal pseudomolecules, including the Z sex chromosome. The mitochondrial genome has also been assembled and is 25.86 kilobases in length. Gene annotation of this assembly on Ensembl identified 13,811 protein coding genes.

## Species taxonomy

Eukaryota; Opisthokonta; Metazoa; Eumetazoa; Bilateria; Protostomia; Ecdysozoa; Panarthropoda; Arthropoda; Mandibulata; Pancrustacea; Hexapoda; Insecta; Dicondylia; Pterygota; Neoptera; Endopterygota; Amphiesmenoptera; Lepidoptera; Glossata; Neolepidoptera; Heteroneura; Ditrysia; Tineoidea; Tineidae; Tineinae; *Tinea*; *Tinea pellionella* (Linnaeus, 1758) (NCBI:txid41014).

## Background

*Tinea pellionella* is a micro-moth in the family Tineidae and is commonly known as the Case-bearing Clothes Moth. It is one of a handful of moths worldwide whose larvae feed on clothing, and is therefore the regular subject of articles in the mass media (e.g. Nast, 2018; Saner, 2019).

The larvae feed on natural fibres which are rich in keratin: mainly wool, fur and feathers, and it can be a serious pest. Although originating in Europe, it can now be found worldwide although it is restricted to temperate and cool Mediterranean climatic zones. It is mainly found indoors, although it has become rarer in the UK with the advent of central heating resulting in warmer and dryer homes ( Gaedike, 2019). It can also be found in outbuildings, warehouses, and occasionally outdoors. Although it is thought to be associated with bird’s nests ( Gaedike, 2019; Plarre & Krüger-Carstensen, 2011), a large study of the moths of bird nests did not find this species ( Boyes, 2018).

The moth is small (forewing length 4–8 mm) with greyish brown forewings. Fresh specimens have up to three spots on each wing, but these are soon worn away in older specimens. In colder buildings it has one generation a year, flying between April and October; however in warmer buildings it can be multi-brooded ( Sterling *et al.*, 2012).

The moth larvae construct a tube from spun silk, supplemented by fibres from their food source. The larva moves around in the tube which acts both as a protective case and as a means of camouflage as the colour of the case reflects that of their food source. Pupation takes place in the case ( Langmaid *et al.*, 2018).

Although some authorities suggest that the moth larvae can feed on plant material (Natural History Museum, no date), feeding experiments demonstrated that *T. pellionella* larvae can only develop on foodstuffs of animal origin. However, its close relative, *Tineola bisselliella* can also feed on plant materials. It was suggested that the reason for this difference is that *T. pellionella* can only metabolise cholesterol, unlike *T. bisselliella* which can use both cholesterol and phytosterols ( Ishii & Kawahara, 1966).

The genome of *T. pellionella* was sequenced as part of the Darwin Tree of Life Project, a collaborative effort to sequence all named eukaryotic species in the Atlantic Archipelago of Britain and Ireland. Here we present a chromosomally complete genome sequence for *Tinea pellionella* based on one female specimen from Wytham Woods, Oxfordshire, UK.

## Genome sequence report

**Figure 1.**
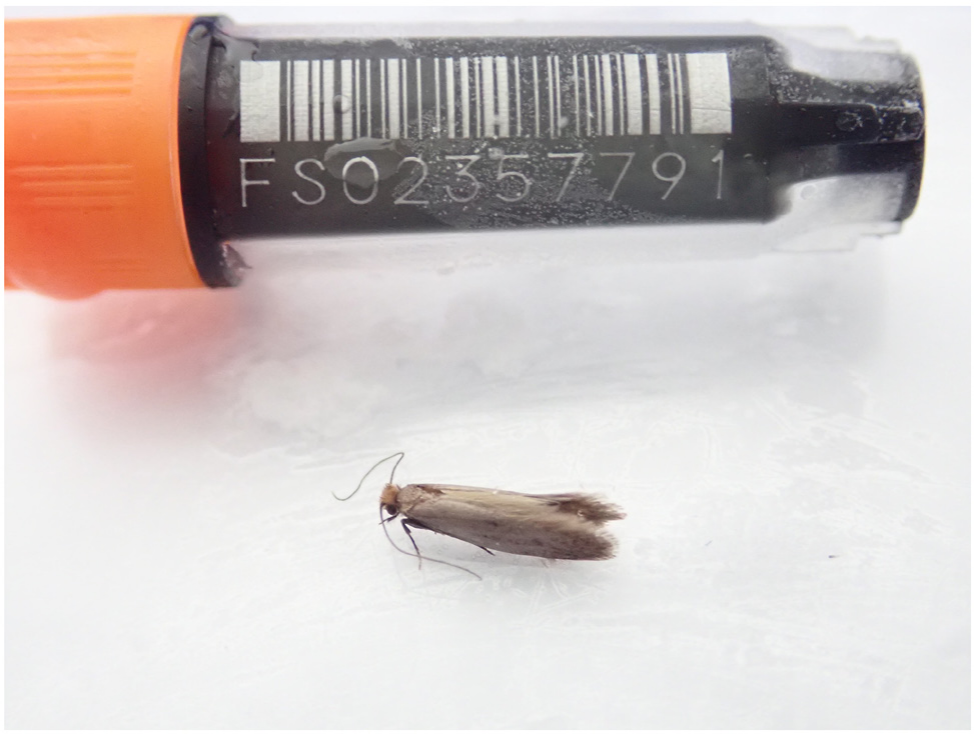
Photograph of the *Tinea pellionella* (ilTinPell1) specimen used for genome sequencing.

The genome was sequenced from one female *Tinea pellionella* ( Figure 1) collected from Wytham Woods, Oxfordshire, UK (51.77, –1.34). A total of 66-fold coverage in Pacific Biosciences single-molecule HiFi long reads was generated. Primary assembly contigs were scaffolded with chromosome conformation Hi-C data. Manual assembly curation corrected 23 missing joins or mis-joins and removed two haplotypic duplications.

**Figure 2.**
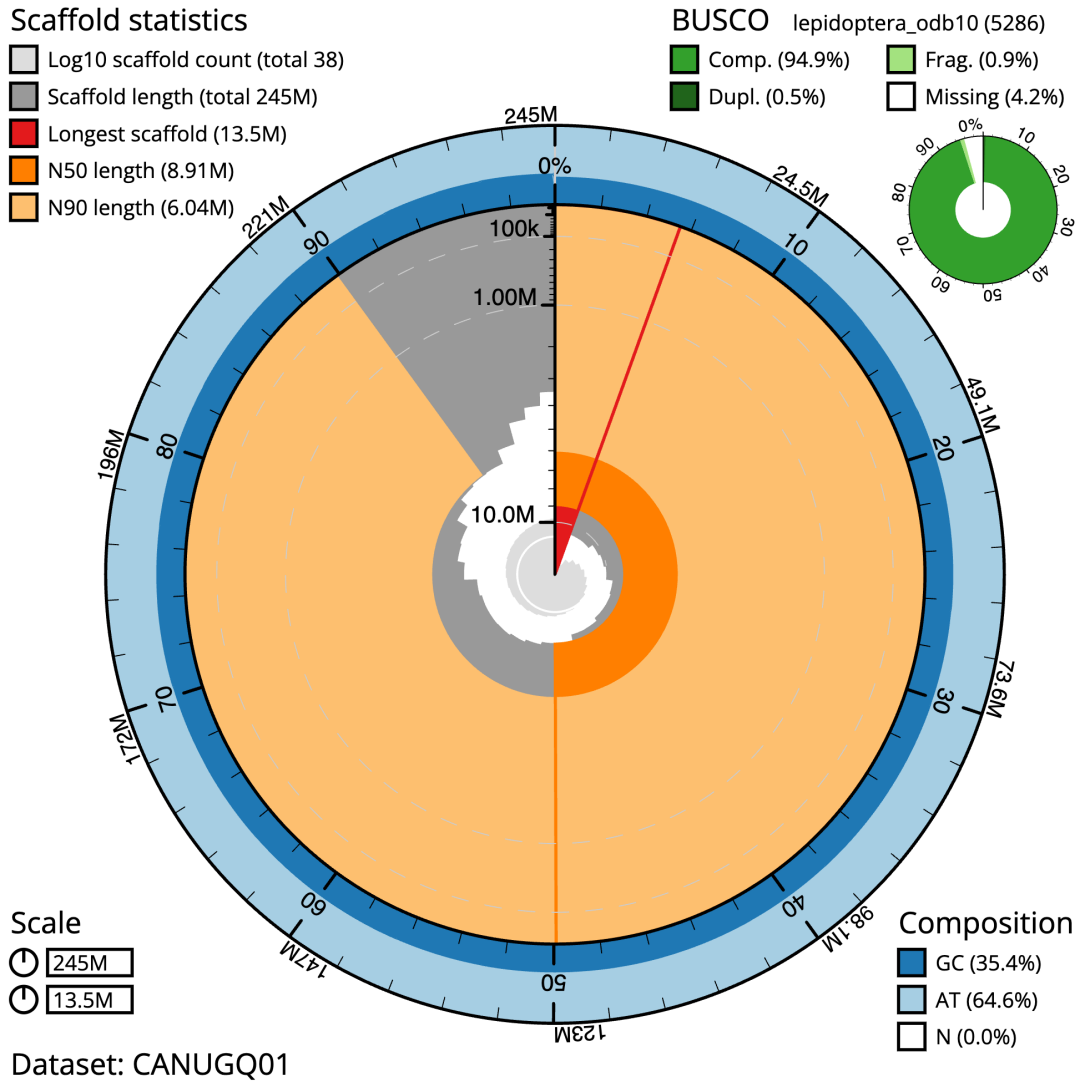
Genome assembly of *Tinea pellionella*, ilTinPell1.1: metrics. The BlobToolKit Snailplot shows N50 metrics and BUSCO gene completeness. The main plot is divided into 1,000 size-ordered bins around the circumference with each bin representing 0.1% of the 245,305,114 bp assembly. The distribution of scaffold lengths is shown in dark grey with the plot radius scaled to the longest scaffold present in the assembly (13,540,534 bp, shown in red). Orange and pale-orange arcs show the N50 and N90 scaffold lengths (8,907,774 and 6,044,544 bp), respectively. The pale grey spiral shows the cumulative scaffold count on a log scale with white scale lines showing successive orders of magnitude. The blue and pale-blue area around the outside of the plot shows the distribution of GC, AT and N percentages in the same bins as the inner plot. A summary of complete, fragmented, duplicated and missing BUSCO genes in the lepidoptera_odb10 set is shown in the top right. An interactive version of this figure is available at https://blobtoolkit.genomehubs.org/view/CANUGQ01/dataset/CANUGQ01/snail.

**Figure 3.**
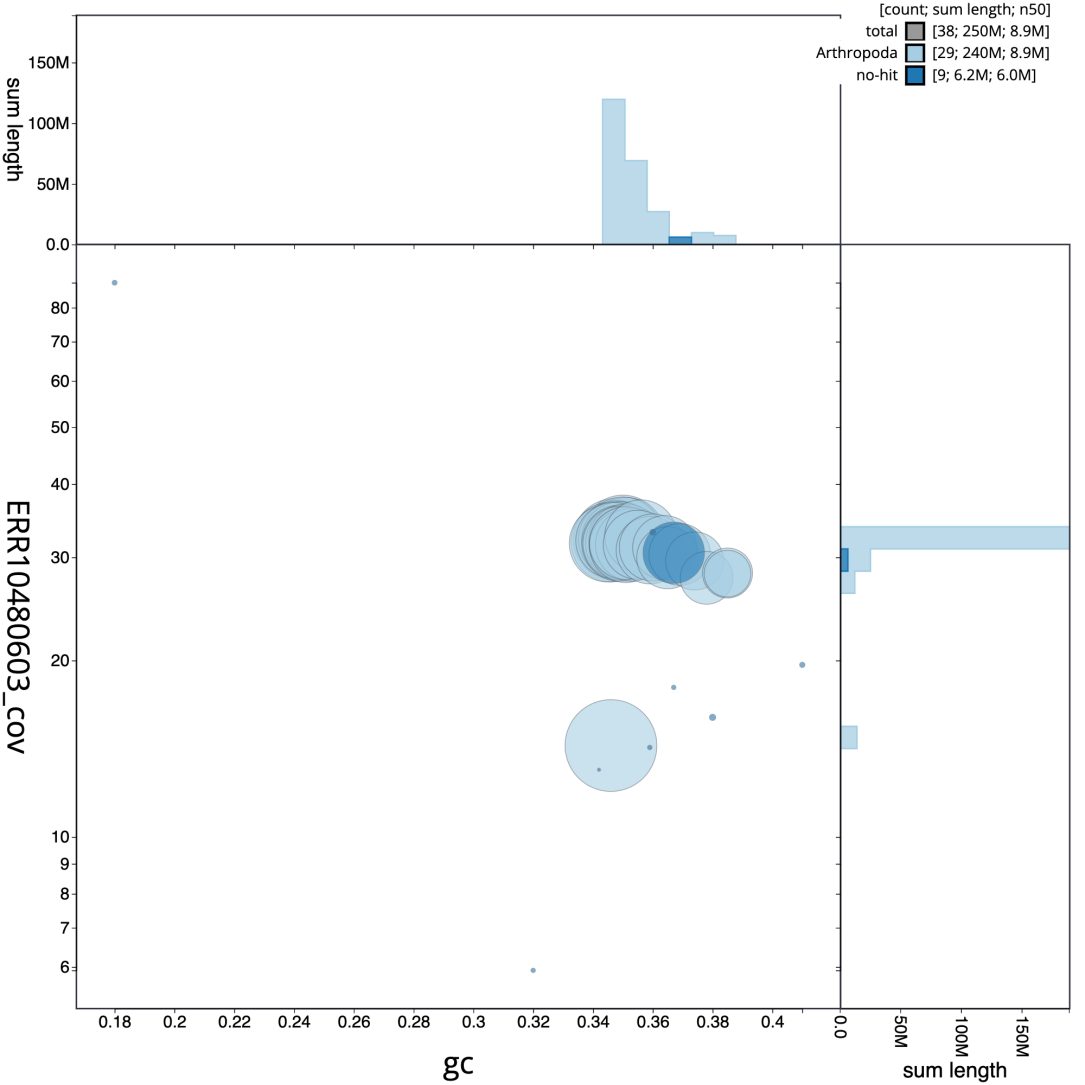
Genome assembly of *Tinea pellionella*, ilTinPell1.1: BlobToolKit GC-coverage plot. Scaffolds are coloured by phylum. Circles are sized in proportion to scaffold length. Histograms show the distribution of scaffold length sum along each axis. An interactive version of this figure is available at https://blobtoolkit.genomehubs.org/view/CANUGQ01/dataset/CANUGQ01/blob.

**Figure 4.**
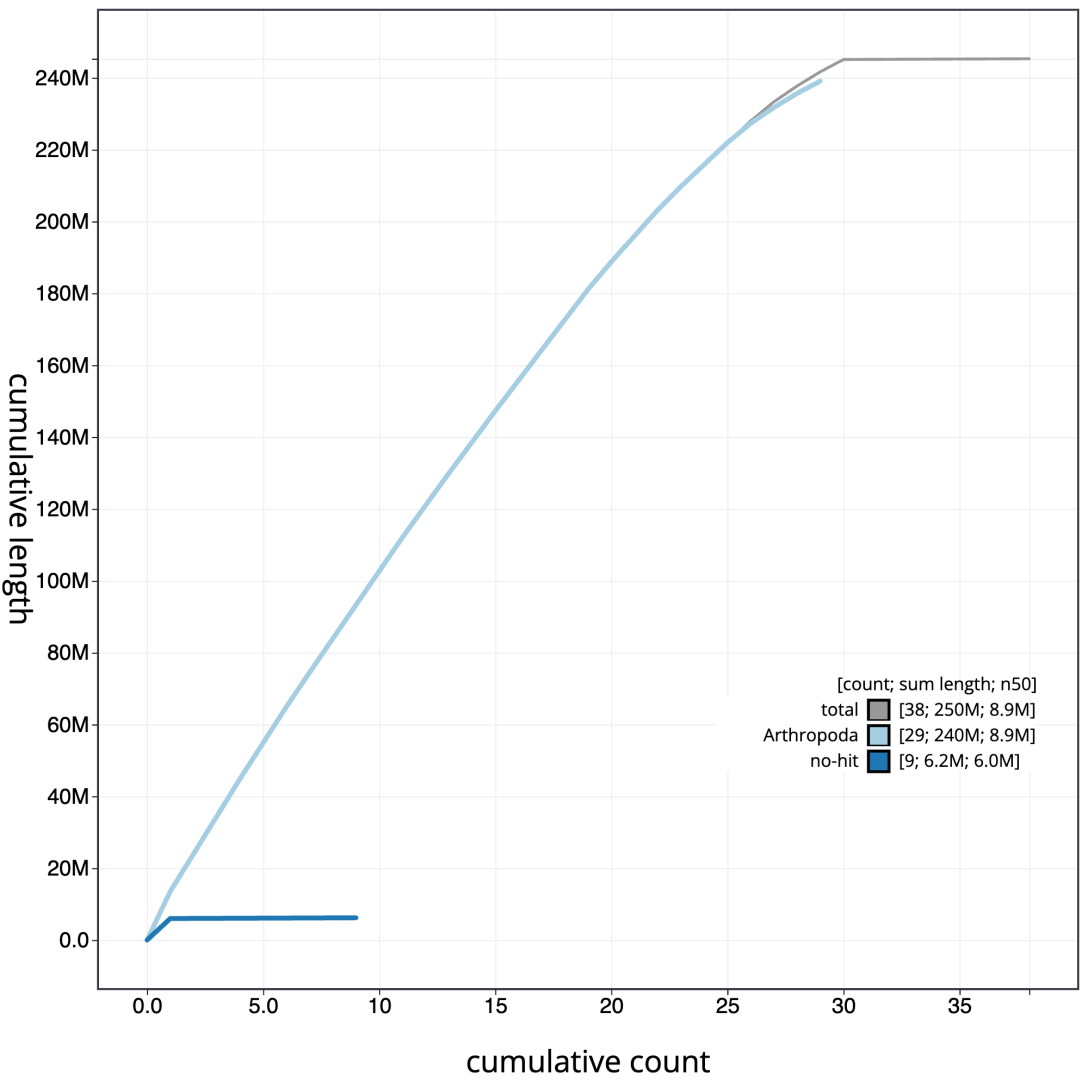
Genome assembly of *Tinea pellionella*, ilTinPell1.1: BlobToolKit cumulative sequence plot. The grey line shows cumulative length for all scaffolds. Coloured lines show cumulative lengths of scaffolds assigned to each phylum using the buscogenes taxrule. An interactive version of this figure is available at https://blobtoolkit.genomehubs.org/view/CANUGQ01/dataset/CANUGQ01/cumulative.

**Figure 5.**
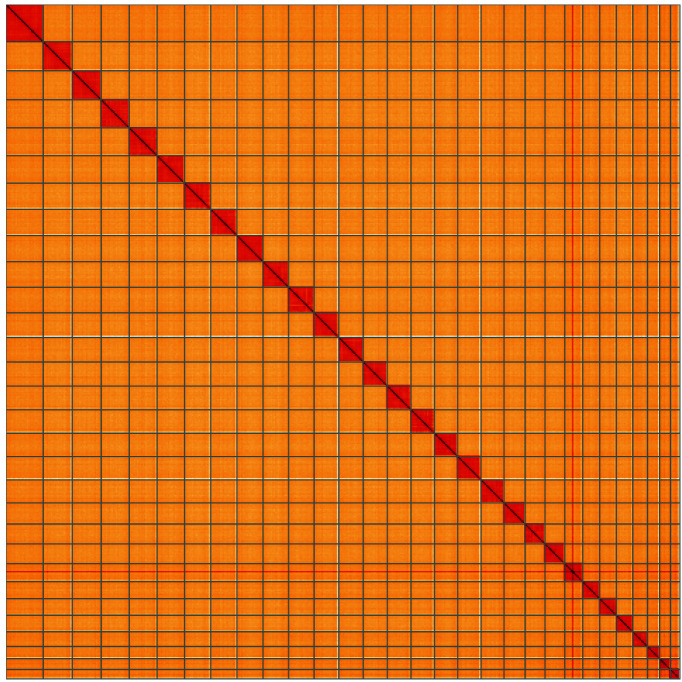
Genome assembly of *Tinea pellionella*, ilTinPell1.1: Hi-C contact map of the ilTinPell1.1 assembly, visualised using HiGlass. Chromosomes are shown in order of size from left to right and top to bottom. An interactive version of this figure may be viewed at https://genome-note-higlass.tol.sanger.ac.uk/l/?d=YjL-3ug3QWuhQuVU_8cmug.

The final assembly has a total length of 245.3 Mb in 37 sequence scaffolds with a scaffold N50 of 8.9 Mb ( Table 1). The snailplot in Figure 2 provides a summary of the assembly statistics, while the distribution of assembly scaffolds on GC proportion and coverage is shown in Figure 3. The cumulative assembly plot in Figure 4 shows curves for subsets of scaffolds assigned to different phyla. Most (99.91%) of the assembly sequence was assigned to 30 chromosomal-level scaffolds, representing 29 autosomes and the Z sex chromosome. The Z chromosome was identified based on coverage; a W chromosome was not identified. Chromosome-scale scaffolds confirmed by the Hi-C data are named in order of size ( Figure 5; Table 2). While not fully phased, the assembly deposited is of one haplotype. Contigs corresponding to the second haplotype have also been deposited. The mitochondrial genome was also assembled and can be found as a contig within the multifasta file of the genome submission.

**Table 1.** Genome data for *Tinea pellionella*, ilTinPell1.1.

Project accession data		
Assembly identifier	ilTinPell1.1	
Species	*Tinea pellionella*	
Specimen	ilTinPell1	
NCBI taxonomy ID	41014	
BioProject	PRJEB57420	
BioSample ID	SAMEA7701521	
Isolate information	ilTinPell1, female: whole organism (DNA sequencing) ilTinPell2, whole organism (Hi-C sequencing)	
Assembly metrics *		*Benchmark*
Consensus quality (QV)	66.5	*≥ 50*
*k*-mer completeness	100.0%	*≥ 95%*
BUSCO **	C:94.9%[S:94.4%,D:0.5%], F:0.9%,M:4.2%,n:5,286	*C ≥ 95%*
Percentage of assembly mapped to chromosomes	99.91%	*≥ 95%*
Sex chromosomes	Z	*localised homologous pairs*
Organelles	Mitochondrial genome: 25.86 kb	*complete single alleles*
Raw data accessions		
PacificBiosciences SEQUEL II	ERR10480603	
Hi-C Illumina	ERR10489908	
Genome assembly		
Assembly accession	GCA_948150575.1	
*Accession of alternate haplotype*	GCA_948150625.1	
Span (Mb)	245.3	
Number of contigs	102	
Contig N50 length (Mb)	4.6	
Number of scaffolds	37	
Scaffold N50 length (Mb)	8.9	
Longest scaffold (Mb)	13.54	
Genome annotation		
Number of protein-coding genes	13,811	
Number of gene transcripts	13,992	

* Assembly metric benchmarks are adapted from column VGP-2020 of “Table 1: Proposed standards and metrics for defining genome assembly quality” from ( Rhie *et al.*, 2021). ** BUSCO scores based on the lepidoptera_odb10 BUSCO set using version 5.3.2. C = complete [S = single copy, D = duplicated], F = fragmented, M = missing, n = number of orthologues in comparison. A full set of BUSCO scores is available at https://blobtoolkit.genomehubs.org/view/CANUGQ01/dataset/CANUGQ01/busco.

**Table 2.** Chromosomal pseudomolecules in the genome assembly of *Tinea pellionella*, ilTinPell1.

INSDC accession	Chromosome	Length (Mb)	GC%
OX411245.1	1	10.54	34.5
OX411246.1	2	10.53	35.0
OX411247.1	3	10.22	34.5
OX411248.1	4	10.1	35.0
OX411249.1	5	9.98	35.0
OX411250.1	6	9.57	34.5
OX411251.1	7	9.5	35.0
OX411252.1	8	9.5	35.0
OX411253.1	9	9.3	34.5
OX411254.1	10	9.29	35.0
OX411255.1	11	8.94	35.0
OX411256.1	12	8.91	35.0
OX411257.1	13	8.76	35.0
OX411258.1	14	8.62	35.0
OX411259.1	15	8.52	35.0
OX411260.1	16	8.49	35.5
OX411261.1	17	8.49	35.0
OX411262.1	18	8.37	35.5
OX411263.1	19	7.64	35.5
OX411264.1	20	7.25	36.0
OX411265.1	21	7.18	36.0
OX411266.1	22	6.55	36.5
OX411267.1	23	6.14	36.5
OX411268.1	24	6.04	37.0
OX411269.1	25	6.01	36.5
OX411270.1	26	5.35	37.5
OX411271.1	27	4.41	38.0
OX411272.1	28	3.94	38.5
OX411273.1	29	3.43	38.5
OX411244.1	Z	13.54	34.5
OX411274.1	MT	0.03	18.0

The estimated Quality Value (QV) of the final assembly is 66.5 with *k*-mer completeness of 100.0%, and the assembly has a BUSCO v5.3.2 completeness of 94.9% (single = 94.4%, duplicated = 0.5%), using the lepidoptera_odb10 reference set ( *n* = 5,286).

Metadata for specimens, barcode results, spectra estimates, sequencing runs, contaminants and pre-curation assembly statistics are given at https://links.tol.sanger.ac.uk/species/41014.

## Genome annotation report

The *Tinea pellionella* genome assembly (GCA_948150575.1) was annotated at the European Bioinformatics Institute (EBI) using the Ensembl rapid annotation pipeline ( Table 1; https://rapid.ensembl.org/Tinea_pellionella_GCA_948150575.1/Info/Index). The resulting annotation includes 13,992 transcribed mRNAs from 13,811 protein-coding genes.

## Methods

### Sample acquisition and nucleic acid extraction

The *Tinea pellionella* specimens used for DNA sequencing (specimen ID Ox000659, ToLID ilTinPell1) and Hi-C sequencing (specimen ID Ox000660, ToLID ilTinPell2) were collected from Wytham Woods, Oxfordshire (biological vice-county Berkshire), UK (latitude 51.77, longitude –1.34) on 2020-07-20 using a light trap. The specimens were collected and identified by Douglas Boyes (University of Oxford) and preserved on dry ice.

The workflow for high molecular weight (HMW) DNA extraction at the Wellcome Sanger Institute (WSI) includes a sequence of core procedures: sample preparation; sample homogenisation, DNA extraction, fragmentation, and clean-up. In sample preparation, the ilTinPell1 sample was weighed and dissected on dry ice ( Jay *et al.*, 2023). Tissue from the whole organism was homogenised using a PowerMasher II tissue disruptor ( Denton *et al.*, 2023a). HMW DNA was extracted using the Automated MagAttract v1 protocol ( Sheerin *et al.*, 2023). DNA was sheared into an average fragment size of 12–20 kb in a Megaruptor 3 system with speed setting 30 ( Todorovic *et al.*, 2023). Sheared DNA was purified by solid-phase reversible immobilisation ( Strickland *et al.*, 2023): in brief, the method employs a 1.8X ratio of AMPure PB beads to sample to eliminate shorter fragments and concentrate the DNA. The concentration of the sheared and purified DNA was assessed using a Nanodrop spectrophotometer and Qubit Fluorometer and Qubit dsDNA High Sensitivity Assay kit. Fragment size distribution was evaluated by running the sample on the FemtoPulse system.

Protocols developed by the WSI Tree of Life laboratory are publicly available on protocols.io ( Denton *et al.*, 2023b).

### Sequencing

Pacific Biosciences HiFi circular consensus DNA sequencing libraries were constructed according to the manufacturers’ instructions. DNA sequencing was performed by the Scientific Operations core at the WSI on a Pacific Biosciences SEQUEL II instrument. Hi-C data were also generated from whole organism tissue of ilTinPell2 using the Arima2 kit and sequenced on the Illumina NovaSeq 6000 instrument.

### Genome assembly, curation and evaluation

Assembly was carried out with Hifiasm ( Cheng *et al.*, 2021) and haplotypic duplication was identified and removed with purge_dups ( Guan *et al.*, 2020). The assembly was then scaffolded with Hi-C data ( Rao *et al.*, 2014) using YaHS ( Zhou *et al.*, 2023). The assembly was checked for contamination and corrected as described previously ( Howe *et al.*, 2021). Manual curation was performed using HiGlass ( Kerpedjiev *et al.*, 2018) and PretextView ( Harry, 2022). The mitochondrial genome was assembled using MitoHiFi ( Uliano-Silva *et al.*, 2023), which runs MitoFinder ( Allio *et al.*, 2020) or MITOS ( Bernt *et al.*, 2013) and uses these annotations to select the final mitochondrial contig and to ensure the general quality of the sequence.

A Hi-C map for the final assembly was produced using bwa-mem2 ( Vasimuddin *et al.*, 2019) in the Cooler file format ( Abdennur & Mirny, 2020). To assess the assembly metrics, the *k*-mer completeness and QV consensus quality values were calculated in Merqury ( Rhie *et al.*, 2020). This work was done using Nextflow ( Di Tommaso *et al.*, 2017) DSL2 pipelines “sanger-tol/readmapping” ( Surana *et al.*, 2023a) and “sanger-tol/genomenote” ( Surana *et al.*, 2023b). The genome was analysed within the BlobToolKit environment ( Challis *et al.*, 2020) and BUSCO scores ( Manni *et al.*, 2021; Simão *et al.*, 2015) were calculated.

Table 3 contains a list of relevant software tool versions and sources.

**Table 3.** Software tools: versions and sources.

Software tool	Version	Source
BlobToolKit	4.2.1	https://github.com/blobtoolkit/blobtoolkit
BUSCO	5.3.2	https://gitlab.com/ezlab/busco
Hifiasm	0.16.1-r375	https://github.com/chhylp123/hifiasm
HiGlass	1.11.6	https://github.com/higlass/higlass
Merqury	MerquryFK	https://github.com/thegenemyers/MERQURY.FK
MitoHiFi	2	https://github.com/marcelauliano/MitoHiFi
PretextView	0.2	https://github.com/wtsi-hpag/PretextView
purge_dups	1.2.3	https://github.com/dfguan/purge_dups
sanger-tol/genomenote	v1.0	https://github.com/sanger-tol/genomenote
sanger-tol/readmapping	1.1.0	https://github.com/sanger-tol/readmapping/tree/1.1.0
YaHS	yahs-1.1.91eebc2	https://github.com/c-zhou/yahs

### Genome annotation

The BRAKER2 pipeline ( Brůna *et al.*, 2021) was used in the default protein mode to generate annotation for the *Tinea pellionella* assembly (GCA_948150575.1) in Ensembl Rapid Release at the EBI.

### Wellcome Sanger Institute – Legal and Governance

The materials that have contributed to this genome note have been supplied by a Darwin Tree of Life Partner. The submission of materials by a Darwin Tree of Life Partner is subject to the **‘Darwin Tree of Life Project Sampling Code of Practice’**, which can be found in full on the Darwin Tree of Life website here. By agreeing with and signing up to the Sampling Code of Practice, the Darwin Tree of Life Partner agrees they will meet the legal and ethical requirements and standards set out within this document in respect of all samples acquired for, and supplied to, the Darwin Tree of Life Project.

Further, the Wellcome Sanger Institute employs a process whereby due diligence is carried out proportionate to the nature of the materials themselves, and the circumstances under which they have been/are to be collected and provided for use. The purpose of this is to address and mitigate any potential legal and/or ethical implications of receipt and use of the materials as part of the research project, and to ensure that in doing so we align with best practice wherever possible. The overarching areas of consideration are:

•      Ethical review of provenance and sourcing of the material

•      Legality of collection, transfer and use (national and international)

Each transfer of samples is further undertaken according to a Research Collaboration Agreement or Material Transfer Agreement entered into by the Darwin Tree of Life Partner, Genome Research Limited (operating as the Wellcome Sanger Institute), and in some circumstances other Darwin Tree of Life collaborators.

## Data Availability

European Nucleotide Archive: *Tinea pellionella* (casemaking clothes moth). Accession number PRJEB57420; https://identifiers.org/ena.embl/PRJEB57420 ( Wellcome Sanger Institute, 2023). The genome sequence is released openly for reuse. The *Tinea pellionella* genome sequencing initiative is part of the Darwin Tree of Life (DToL) project. All raw sequence data and the assembly have been deposited in INSDC databases. Raw data and assembly accession identifiers are reported in Table 1.

## Author information

Members of the University of Oxford and Wytham Woods Genome Acquisition Lab are listed here: https://doi.org/10.5281/zenodo.7125292.

Members of the Darwin Tree of Life Barcoding collective are listed here: https://doi.org/10.5281/zenodo.4893703.

Members of the Wellcome Sanger Institute Tree of Life Management, Samples and Laboratory team are listed here: https://doi.org/10.5281/zenodo.10066175.

Members of Wellcome Sanger Institute Scientific Operations: Sequencing Operations are listed here: https://doi.org/10.5281/zenodo.10043364.

Members of the Wellcome Sanger Institute Tree of Life Core Informatics team are listed here: https://doi.org/10.5281/zenodo.10066637.

Members of the Tree of Life Core Informatics collective are listed here: https://doi.org/10.5281/zenodo.5013541.

Members of the Darwin Tree of Life Consortium are listed here: https://doi.org/10.5281/zenodo.4783558.
