# Supplementary material for: The genome sequence of the Case-bearing Clothes moth, Tinea pellionella (Linnaeus, 1758)
Source: Wellcome Open Res. 2024 Nov 4;9:119. Originally published 2024 Mar 1. [Version 2] doi: 10.12688/wellcomeopenres.21015.2 (PMC11530743; doi:10.12688/wellcomeopenres.21015.2)
Supplement: Peer review history

# Reviewer report: Reviewer response for version 2

**DOI:** 10.21956/wellcomeopenres.25758.r109699

**Published:** 2024-11-12

**Authors:** Valentina Todisco

**Affiliations:** 1 Paris Lodron University of Salzburg, Salzburg, Austria

I think that the article can be now accepted for the Indexing.

Are sufficient details of methods and materials provided to allow replication by others?

Yes

Is the rationale for creating the dataset(s) clearly described?

Yes

Are the datasets clearly presented in a useable and accessible format?

Yes

Are the protocols appropriate and is the work technically sound?

Yes

Reviewer Expertise:

Entomology. Lepidoptera, Molecular Biology

I confirm that I have read this submission and believe that I have an appropriate level of expertise to confirm that it is of an acceptable scientific standard.

# Reviewer report: Reviewer response for version 2

**DOI:** 10.21956/wellcomeopenres.25758.r109701

**Published:** 2024-11-05

**Authors:** Jonathan Badger

**Affiliations:** 1 National Cancer Institute, Bethesda, Maryland, USA

I have no further comments to make on the new revision

Are sufficient details of methods and materials provided to allow replication by others?

Yes

Is the rationale for creating the dataset(s) clearly described?

Yes

Are the datasets clearly presented in a useable and accessible format?

Yes

Are the protocols appropriate and is the work technically sound?

Yes

Reviewer Expertise:

My research is on the computational side of genomics, currently primarily on humans, mice, and their microbiomes, but I have also worked (and continue to work on the side) on insects.

I confirm that I have read this submission and believe that I have an appropriate level of expertise to confirm that it is of an acceptable scientific standard.

# Reviewer report: Reviewer response for version 1

**DOI:** 10.21956/wellcomeopenres.23250.r103943

**Published:** 2024-11-01

**Authors:** Sarah Inwood

**Affiliations:** 1 University of Otago, Otago, New Zealand

The authors sequenced and assembled a chromosomal scale genome assembly for the case-bearing clothes moth *Tinea pellionella*. This assembly used HiFi long reads and Hi-C data, resulting in a high-quality nuclear genome assembly, alongside a mitochondrial genome assembly. 

- BRAKER2 and bwa-mem2 were used but no versions were specified in the software tools table.
- The method for mitochondrial genome assembly could be clarified, as the authors mention using MitoHiFi, which can use MitoFinder or MITOS but do not report which one was used for their assembly.
- The identification of the Z chromosome based on coverage could be explained further. The fact that the W chromosome was not identified in the assembly, despite the sample being female, could also benefit from further discussion. Potential reasons for this could have been suggested e.g. how confident is the assignment of the sample as female, are there reasons in the assembly and curation pipeline that it might not have been assembled/retained?

Are sufficient details of methods and materials provided to allow replication by others?

Partly

Is the rationale for creating the dataset(s) clearly described?

Yes

Are the datasets clearly presented in a useable and accessible format?

Yes

Are the protocols appropriate and is the work technically sound?

Yes

Reviewer Expertise:

Genomics, host-parasite interactions, parasitoid wasps, insects, viruses, biocontrol

I confirm that I have read this submission and believe that I have an appropriate level of expertise to confirm that it is of an acceptable scientific standard, however I have significant reservations, as outlined above.

# Reviewer report: Reviewer response for version 1

**DOI:** 10.21956/wellcomeopenres.23250.r103938

**Published:** 2024-10-24

**Authors:** Jonathan Badger

**Affiliations:** 1 National Cancer Institute, Bethesda, Maryland, USA

This is a well written and straightforward genome manuscript; the authors provide sufficient detail as to the sequencing and bioinformatics methods used. I checked and the data is indeed deposited in the ENA as stated.

 My only real concern with the manuscript is the comment without explanation that while the Z chromosome was found, the W was not. As the source of the DNA is a female moth, both should be present.  Can the authors maybe add some explanation in the manuscript as to why it might not have been sequenced?

Are sufficient details of methods and materials provided to allow replication by others?

Yes

Is the rationale for creating the dataset(s) clearly described?

Yes

Are the datasets clearly presented in a useable and accessible format?

Yes

Are the protocols appropriate and is the work technically sound?

Yes

Reviewer Expertise:

My research is on the computational side of genomics, currently primarily on humans, mice, and their microbiomes, but I have also worked (and continue to work on the side) on insects.

I confirm that I have read this submission and believe that I have an appropriate level of expertise to confirm that it is of an acceptable scientific standard.

# Reviewer report: Reviewer response for version 1

**DOI:** 10.21956/wellcomeopenres.23250.r91359

**Published:** 2024-08-06

**Authors:** Valentina Todisco

**Affiliations:** 1 Paris Lodron University of Salzburg, Salzburg, Austria

In this manuscript, the authors present a genome assembly from an individual female *Tinea Pellionella*, a micro-moth commonly known as the Case-bearing Clothes Moth. It is often subject of articles in the mass media because it is a serious pest and it is can now be found worldwide. This study represents therefore an important step in the future control of this species.

**Background**

 It represents an accurate description of the ecology and life cycle of the species.

- The first two sentences of the fifth paragraph need references.

**Genome sequence report and Methods**

 They are clearly described and they follow the standard procedure in Wellcome Sanger Institute (WSI).

Are sufficient details of methods and materials provided to allow replication by others?

Yes

Is the rationale for creating the dataset(s) clearly described?

Yes

Are the datasets clearly presented in a useable and accessible format?

Yes

Are the protocols appropriate and is the work technically sound?

Yes

Reviewer Expertise:

Entomology. Lepidoptera, Molecular Biology

I confirm that I have read this submission and believe that I have an appropriate level of expertise to confirm that it is of an acceptable scientific standard, however I have significant reservations, as outlined above.
